# The emotional impact of urinary tract infections in women: a qualitative analysis

**DOI:** 10.1186/s12905-022-01757-3

**Published:** 2022-05-18

**Authors:** Larissa Grigoryan, Aruni Mulgirigama, Marcy Powell, Guido Schmiemann

**Affiliations:** 1 Department of Family and Community Medicine, Baylor College of Medicine, Houston, TX USA; 2 Global Specialty and Primary Care, GlaxoSmithKline, Brentford, Middlesex, UK; 3 Safety and Medical Governance, GlaxoSmithKline, Research Triangle, NC USA; 4 Department for Health Services Research, Institute of Public Health and Nursing Research, University of Bremen, Bremen, Germany

**Keywords:** Cost of illness, Cystitis, General practice, Patient preference, Quality of life, Treatment failure, Urinary tract infection

## Abstract

**Background:**

While many studies address the clinical management of participants with uncomplicated urinary tract infection (uUTI), the emotional impact of uUTIs has been investigated less often. The aim of this qualitative study was to understand the emotional experience of women with uUTIs.

**Methods:**

This was a qualitative, exploratory, in-depth interview-based study conducted among women in the United States (US) and Germany. Women aged ≥ 18 years with at least one uUTI treated with antibiotics in the past year were recruited through a patient community panel and physician referrals. Participants were recruited using purposive sampling to include an equal split of those with 1 or ≥ 2 antibiotics, and an equal split of those treated for a single or recurrent uUTIs (≥ 2 uUTIs in the past year). A structured telephone interview included questions about symptoms, diagnosis, treatment, and retreatment (if any). Each participant was queried about her emotions and the impact of the uUTI on life activities. Thematic analysis of responses was carried out to identify common themes.

**Results:**

A total of 65 participants completed the interview, 40 (61.5%) from the US and 25 (38.5%) from Germany. Major themes that emerged from the analyses included (1) a wide range of negative emotions were experienced due to uUTI symptoms, interference with activities of daily life, and effects on relationships and sleep; (2) varied emotions and understanding related to uUTI treatment and management approaches; (3) treatment failure caused frustration, worry, and anger; and (4) the prospect of recurrent uUTIs provoked dread and helplessness.

**Conclusion:**

Our research uncovered emotions of helplessness and dread experienced by women in the context of uUTI clinical treatment failure and recurrent uUTIs. Knowing patients’ perspectives on UTI management will help guide the development of patient education and improve shared decision-making.

**Supplementary Information:**

The online version contains supplementary material available at 10.1186/s12905-022-01757-3.

## Background

Urinary tract infection (UTI) is the most common bacterial infection presenting in outpatient settings [1]. Most UTIs occur in otherwise healthy women without symptoms of upper urinary tract or systemic infection and are considered uncomplicated UTIs (uUTIs) [2]. Unfortunately, many women experience recurrent uUTIs, which can be defined as at least two episodes within 6 months or at least three episodes within 1 year [3–5]. The rate of recurrent UTIs in women varies from 15 to 50% across studies, depending on the patient population studied and definition used [3, 4, 6–9].

While many studies address the clinical management of patients with uUTIs, the emotional impact of uUTIs has been investigated less often. Few studies using self-report measures have investigated the impact of uUTIs on patients’ quality of life and emotional and mental health [10–12]. In a survey of 1941 women from five European countries, participants with recent acute UTI had lower physical and mental health scores than the norms obtained from the 2009 general United States (US) population [11]. Similarly, an international study of 575 patients in seven countries found a reduction in UTIs correlated with a reduction in anxiety and depression scores [12]. While these studies demonstrate the negative impact of uUTIs, the use of standardized quantitative measures fails to convey the specific emotions experienced by women with uUTIs.

Eriksson et al. [13] conducted a qualitative interview study to characterize the emotional experiences of 20 women aged 67–96 years who had suffered from recurrent uUTIs. These women described a range of negative emotional responses to the experience of uUTIs, including anxiety, depression, dejection, and surliness, as well as challenges in obtaining needed relief [13]. The richness of the emotional experience of women with uUTIs from these findings suggests the need to better understand patients’ emotional reactions to this health challenge, an understanding that can inform patient education to address women’s fears and promote shared decision-making. The aim of this qualitative study was to understand the emotional experience and impact of uUTIs in women.

## Methods

This was a qualitative, exploratory, in-depth interview-based study conducted by Adelphi Research between November 26 and December 18, 2019. An interview-based methodology was selected to allow for a more in-depth discussion and to pose a lower threshold for participants to report private or intimate details compared with focus groups. As part of these interviews, a semi-structured narrative self-task component was used, via the SoGoSurvey mobile app platform, as an additional help and source of information to support and engage participants throughout the interview [14]. Three tasks were set over the course of the week, describing what it’s like to live with a uUTI; selecting images to represent how it feels to experience a uUTI; and recording top three struggles when experiencing a uUTI. The app added value for the interviews as it provided a reference point for describing the emotional impact of UTI, such as images to help participants to represent their feelings. This was followed by semi-structured interviews, whereby respondents recalled their own uUTI experience, was used to identify and explore emotions that are previously undefined. Repeat interviews were not carried out although the two stages of data collection took place on separate dates. Recruitment agencies offered support with onboarding, including technical support and explaining how the data will be used, to offer reassurance. All research methods were conducted in accordance with all national and international data protection laws and relevant industry guidelines including specific and/or local regulations such as Arbeitskreis Deutscher Markt- und Sozialforschungsinstitute or Bundesverband der Marktforschung in Germany, British Healthcare Business Intelligence Association, European Society for Opinion and Marketing Research, and European Pharmaceutical Market Research Association (EphMRA). General Data Protection Regulation guidelines were followed to ensure full patient data confidentiality.

### Recruitment process

The recruitment process included a patient community panel, such as email and social media, and physician referrals. Referring physicians were directed to identify participants according to the following definition of uUTI: “a woman with uncomplicated UTI presents with local signs and symptoms such as dysuria, frequency, urgency, or lower abdominal/suprapubic pain. The patient, otherwise healthy, does not have an anatomical or physiological abnormality of the urinary tract and no systemic signs of infection such as fever (greater than 38 °C) or costovertebral pain.” Women with a fever were excluded as this would indicate complicated UTI (pyelonephritis) [2]. Participants with recurrent or sporadic UTI were included, with specific screening criteria used to ensure capture of both groups. Interested participants contacted the local agencies by phone or email.

The eligibility of interested participants was confirmed via phone. All participants were ≥ 18 years of age, were informed of all rights pertaining to this study, and written informed consent was obtained. Respondents who were recruited via phone were sent a consent to participate form, which was signed and returned to the recruitment agency. This study was non-clinical and was for patient insight purposes only. It was exempt from Institutional Review Board (IRB) requirements per criteria listed in 45 CFR 46.101(b), category 2 for participants from the US, as it used interview procedures recorded in a way that human subjects could not be identified. IRB approval was also not required nor sought for participants from Germany, per the Association of Medical Ethics Committees (AKEK), and the study did not meet the requirements for needing ethical approval, per section 1.3 of the EphMRA guidelines. Respondents were not classified as vulnerable, nor would participation induce undue psychological stress or anxiety.

### Participants

Included participants were required to be female, aged ≥ 18 years, have experienced at least one uUTI in the past year, and have received antibiotic treatment for a uUTI in the past year. Participants must have experienced at least one of the following symptoms during a uUTI episode within the past year: needing to urinate suddenly, needing to urinate more often than usual, or pain or a burning sensation when urinating. We used a purposive sampling approach [15, 16] and stratified participants according to country of origin (US or Germany). Participants were further stratified by the number of antibiotic courses required for a single UTI episode. We estimated that 12 participants per stratum would be sufficient to achieve data saturation [17]. Therefore, a minimum of 12 participants were included in each stratum (for number of antibiotics) within each country (a minimum of 24 participants per country). A minimum of 50% of participants selected globally had experienced recurrent uUTIs.

### Study design and measures

Adelphi Research personnel were responsible for the design of the project, data collection, analysis, and reporting. Adelphi Research are ISO certified and all research was completed in line with ISO20252 (international quality standard for market, opinion, and social research, including insights and data analytics). Adelphi was selected through a competitive bidding process. Adelphi’s proposal was deemed the best option for the work as well as having industry experience in completing work used for medical publication. Participants were recruited through independent healthcare fieldwork agencies in the US and Germany, Schlesinger (US [18] and Searchlight [19]). These agencies were selected as they have gone through a comprehensive supplier assessment process and the necessary technical and organizational measures. These agencies were thoroughly briefed, detailing study objectives, inclusion criteria, and other logistical aspects, followed by subsequent monitoring of the process.

During the screening call, participants were asked to report their age group, gender, the timing of their most recent uUTI, the number of uUTIs experienced in the past year, the number of antibiotic courses received for treatment of each uUTI in the past year, and whether they had experienced any of six specified symptoms during a uUTI episode in the past year.

For eligible participants, a structured, in-depth telephone interview (~ 30 min in duration) was conducted by three female external moderators trained in qualitative interviewing in accordance with the interview format developed by Adelphi Research (see Additional file 1 for text of structured interview, including instructions to the moderator conducting the interview, and see Disclosures section for further information on the external moderators). Interviews were initially pilot tested (N = 5), to ensure that materials were appropriate, and insights captured met the study objectives. As the questionnaire and materials were deemed suitable, with no amendments required, the results from the pilot questionnaires were included in the final sample. Interviews were completed in a setting of the respondent’s choosing. Only the external moderator and the participant were actively conversing during the telephone interviews, although in some cases Adelphi and/or the study sponsor (GlaxoSmithKline plc.) listened on a muted line, with the participant’s prior permission, to ensure that the objectives were met. Throughout the interview, the participant was queried about her emotions and the impact of the uUTI and treatment on life activities. Financial compensation was provided to participants in accordance with local regulations following the completion of the interview. Moderators were independent and had no prior relationship with the participants. Participants were aware that the researcher was employed as an external moderator by GlaxoSmithKline plc. to conduct the study but were not made aware of the moderator’s personal characteristics.

### Analysis of interview texts

Following completion of the interviews, analysts specializing in qualitative healthcare research transcribed key portions of the recordings on an individual participant basis into an analysis grid developed by Adelphi Research, without the use of data management software. According to the biopsychosocial model proposed by Engel in 1977 and elaborated in the years since [20, 21], the participant’s experience of disease includes physical manifestations, psychological sequelae, and impacts in the social context. Drawing on this theory, analysis of the interview texts focused on participants’ experience of physical symptoms and treatment, the impact of uUTI on life activities and relationships, and resulting emotional impacts. Transcripts were not available to participants post-interview, and participants did not provide feedback on findings.

Thematic analysis of responses was carried out by three members of the Adelphi Research team to identify common themes. Member checking was adopted throughout the interview in order to confirm presented themes were accurate. An inductive approach was used to ensure that the full range of participants’ experiences was captured, and insights were not lost by prematurely narrowing the frame of reference. This process involved (1) fully reviewing the interview data, (2) developing codes that best described and represented the data, (3) identifying patterns and common themes among the data, (4) defining themes to best represent the data, and (5) developing the study report to include the thematic story of the findings with detailed and nuanced representation of the data.

When quoting specific participants in this paper, an identification number (e.g., US10) is used that consists of the participant’s country of residence (US or DE [Germany]) and a unique, anonymized participant number within that country.

## Results

### Inclusion and exclusion of participants

In the US, 631 people expressed interest in the study. Of these, 564 did not meet inclusion criteria, 15 withdrew from the study after being recruited due to personal reasons, and 12 did not attend the scheduled interview. In Germany, 90 people were contacted, of whom 45 did not meet inclusion criteria, five did not want to download an app required for an associated mobile ethnography exercise, and 15 did not meet quota criteria. A total of 65 participants completed the in-depth telephone interview, 61.5% from the US and 38.5% from Germany.

### Participant disposition and characteristics

Participant characteristics are summarized in Table 1.

**Table 1:** Participant characteristics

Characteristic, n (%)	United States (n = 40)	Germany (n = 25)	Total (N = 65)
Age, years			
18–29	8 (20.0)	4 (16.0)	12 (18.5)
30–39	6 (15.0)	13 (52.0)	19 (29.2)
40–49	9 (22.5)	8 (32.0)	17 (26.2)
50–59	9 (22.5)	0	9 (13.8)
60–69	5 (12.5)	0	5 (7.7)
≥ 70	3 (7.5)	0	3 (4.6)
Timing of most recent uUTI			
Currently have a uUTI	2 (5.0)	2 (8.0)	4 (6.2)
< 3 months ago	17 (42.5)	15 (60.0)	32 (49.2)
3–6 months ago	6 (15.0)	6 (24.0)	12 (18.5)
7–12 months ago	15 (37.5)	2 (8.0)	17 (26.2)
No. of uUTIs in past year			
≤ 2	21 (52.5)	9 (36.0)	30 (46.2)
> 2	19 (47.5)	16 (64.0)	35 (53.8)
No. of antibiotic treatments required for any uUTI in past year			
1 course for each uUTI	21 (52.5)	12 (48.0)	33 (50.8)
> 1 course for ≥ 1 uUTI	19 (47.5)	13 (52.0)	32 (49.2)
Symptoms experienced with uUTI in past year			
Needing to urinate more often than usual	37 (92.5)	22 (88.0)	59 (90.8)
Needing to urinate suddenly	32 (80.0)	18 (72.0)	50 (76.9)
Pain when urinating	30 (75.0)	23 (92.0)	53 (81.5)
Burning sensation when urinating	31 (77.5)	19 (76.0)	50 (76.9)
Blood in urine	8 (20.0)	1 (4.0)	9 (13.8)
Other^a^	4 (10.0)	0	4 (6.2)

*uUTI* uncomplicated urinary tract infection

^a^Other symptoms reported included discomfort, feeling unwell, disturbed sleep, stomach cramps, pressure when urinating, and not being able to urinate (some participants experienced more than one of these symptoms)

Of the women interviewed, 73.8% were between 18 and 49 years of age. Most participants either had a uUTI at the time of the interview or had experienced one within the past 3 months. Similar proportions of participants had experienced either less than three or three or more uUTIs in the past year. Approximately half of the participants had received one course of antibiotic treatment for each uUTI experienced in the past year.

### Domains and major themes in the emotional experience of uUTI

Analysis of the interview texts focused on three domains of participants’ statements about their uUTI journey: (1) experience of physical symptoms, diagnosis, and treatment; (2) impact of uUTI on life activities and relationships; and (3) the emotional impact of these experiences (Table 2).

**Table 2:** Analysis of interview texts

	Daily activities	Relationships	Finances	Sleep
Participant experience	Repeated visits to toilet Pain/discomfort Need for visits to doctor/pharmacy Need to constantly be in or close to a bathroom	Tiredness Odor Pain Irritability	Cost of HCP consultation Cost of medication Cost of over-the-counter remedies Loss of earnings	Constant waking to go to the bathroom throughout the night Pain/burning disrupting sleep
Impact on life activities	Inability to go about daily duties (e.g., college, work, family life) Disruption to daily routine	Avoidance of intimacy Not wanting to tell others Avoiding social activities Cancelling plans	May have to prioritize which expenditures are essential versus nice to have	Tiredness Irritability Low motivation Withdrawal
Predominant emotions	Frustration Helplessness Loss of control	Isolation Embarrassment	Worry about being able to cover other living costs	Frustration Struggling to remain awake Fear of letting others down

The analysis of interview texts focused on three domains of participants’ uUTI journeys: experience of physical symptoms, diagnosis, treatment, presentation, and failure; impact of uUTI on life activities; and the resulting emotional impact of these experiences

*HCP* healthcare provider, *uUTI* uncomplicated urinary tract infection

**Fig. 1:**
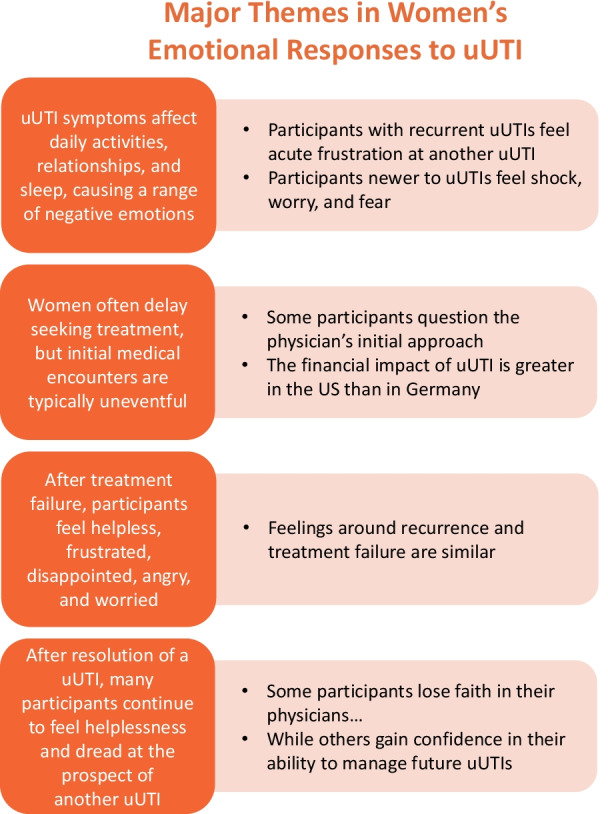
Themes emerging from analysis of interview texts. *uUTI* uncomplicated urinary tract infection

We estimate saturation was achieved after analysis of 20 US participant interviews and 17 German participant interviews. High levels of consistency were observed across the sample, regardless of whether the participant was from the US or Germany, with the exception of the financial impact. Where applicable, differences by country, demography, or clinical profile have been noted in the report of findings. Major themes that emerged from the inductive analyses are summarized in Fig. 1 and described in detail below.

### Wide range of negative emotions due to uUTI symptoms

#### Interference with life activities

Symptoms of uUTI affected multiple areas of daily life, including activities, relationships, and sleep (Table 2). Participants described missing work, study, family, and social commitments, which caused significant disruption. Bathroom stops interfered with daily routines of work, chores, and childcare. Participants also experienced reduced effectiveness at tasks because of pain. One participant said, “Pain kept me from functioning fully in everyday life” (DE18).

Participants were frustrated by the interference of uUTI symptoms with day-to-day life. The continuing need to be close to a bathroom led to difficulty in keeping up with day-to-day activities and therefore frustration and anxiety, as well as embarrassment (especially if “caught short,” as one participant put it). Taking time away from school and work led to high levels of stress and frustration. Impacts on normal routines (e.g., childcare) led to feelings of helplessness due to lack of control.

Regarding experiences at work, participants described frequent toilet visits, vigilance to ensure a bathroom was nearby, and worry about odor associated with the infection. A participant said, “You’re thinking, ‘What are [my coworkers] thinking? They can totally smell this’ … It’s kind of like social uncomfortableness … Sometimes I’ll go to another floor in the building where I think I won’t see anybody I know” (US19).

Difficulty in visiting the doctor for treatment also affected daily life and caused stress and anxiety. Some participants expressed irritation at trying to obtain a doctor’s appointment and having to spend time in the waiting room. One participant said, “Going to the doctor makes me very stressed out and irritated, as it is so time consuming” (US14). Many participants expressed a desire for a more efficient treatment process.

#### Effects on relationships and sleep

uUTIs also caused emotional impacts in the context of relationships. Participants often felt unable and unwilling to involve or share their experience with others, reducing contact and intimacy. The need for frequent bathroom stops when seeing friends resulted in the cancellation of plans, and difficulties in socializing led to withdrawal and loneliness. Participants’ low mood also led to negative emotions around interacting with others and again resulted in feelings of isolation. One woman said, “When I have a uUTI it’s like I wake up on the wrong side of the bed every day” (US38).

Participants described negative impacts on romantic relationships and intimacy because low mood caused irritation. Abstaining from sex, based on a doctor’s advice or symptoms, led to frustration and anxiety. Some participants also mentioned anxiety or the partner mistakenly feeling they are at fault.

Finally, disruption of sleep due to discomfort and getting up during the night resulted in tiredness, irritability, and low motivation, which led to an inability to accomplish day-to-day chores and significant decreases in overall productivity. These impacts on productivity caused feelings of frustration and helplessness. Participants also described irritability due to lack of sleep, which affected relationships with others and led to withdrawal. One participant said, “The sleeplessness is a major problem that affects my ability to function in everyday life” (DE18).

#### Uncomplicated UTI experienced as an acute, high-impact condition

Participants compared the severity of uUTI symptoms to that of other common, acute, high-impact conditions that are quickly resolved but may recur, such as a cold or toothache. The experience appeared to be low in severity but high in impact. One participant expressed, “It’s similar to having flu or a cold—a common illness that many of my friends have had and one that is treatable” (DE25). Another participant stated, “You know when you know you have a toothache coming? I know if I don’t do something now about it, in about four to five days it’s going to be killing me. That’s how I can relate to it sometimes: I know it’s coming, and if I don’t do something now, I’m going to really regret it” (US22).

#### Reaction to recurrent and first-time uUTI

Many participants who experienced recurrent uUTIs felt an acute sense of frustration. When symptoms emerged, they quickly recognized they were experiencing a uUTI. These participants were aware of the uUTI process and impact and therefore tended to see the uUTI as an annoyance or frustration. Because of a lack of awareness or expectation of what was happening and what was to come, participants who were newer to uUTIs tended to feel more shock, worry, and fear in response to initial symptoms than participants who had experienced more uUTIs. One participant said, “I woke up because of the pain, and to start with I didn’t know what was wrong because I had never had this before” (US40). However, many participants who had not experienced recurring uUTIs still had past experience or an awareness of uUTIs through talking to friends and family to draw on.

### Varied experiences with the initial medical encounter

#### Delays in seeking medical attention

Few participants sought medical attention as soon as symptoms started. Barriers to seeking help included the inconvenience or necessity of taking time off work to see a doctor, wanting to avoid the frustration of a doctor’s visit, wanting to solve the problem themselves, hoping the condition would resolve without antibiotics, and reluctance to take antibiotics unless really needed. Some participants in the US referred to cost of care as a barrier to seeking medical attention. One participant said, “I wish I could have a medication on hand, instead of waiting long hours for the doctor” (DE02). A few participants, typically those experiencing their first uUTI, mentioned embarrassment as a barrier to seeking care. Common triggers that ultimately prompted participants to seek help were symptoms that did not go away after a few days, pain that worsened to the point of being unbearable, or actively taking a test strip that came back positive.

#### Financial impact of uUTIs differs in Germany versus US

Based on participants’ accounts, the financial impact of having a uUTI differed for participants in Germany and those in the US. Whilst high levels of consistency were observed between participant responses to the emotional impact of a uUTI, the financial impact upon the participant was the key difference between participants from the US and those from Germany. While out-of-pocket healthcare costs are limited in Germany, multiple participants expressed irritation at having to spend money on a uUTI, particularly when purchasing homeopathic treatments. One participant said, “It is difficult to go the doctor, time-consuming and so on, so sometimes I try to treat it with products that I can buy at a pharmacy, and that can cost quite a lot” (DE19).

For some participants in the US, the financial impact was perceived as irritating. One woman explained, “It’s $35–$40 every time you get a uUTI. It’s a little ridiculous for something supposedly one in three women experience multiple times a year” (US36). However, for other participants in the US, the financial burden was much greater. Missing work to attend a doctor’s appointment was sometimes difficult if the participant did not have sick leave. Out-of-pocket payment for a doctor’s visit and treatment, particularly for participants who were uninsured, meant difficulty paying other bills and led to stress and frustration with the financial shortfall. One participant stated, “I end up having to miss out on work [to attend a medical appointment]. Because I am paycheck to paycheck, I fall behind. And it has a snowball effect for me. It’s frustrating … Eventually I will catch up on my bills, but I do fall behind” (US21).

### Varied emotions and understanding related to uUTI treatment and management approaches

#### Participants may use their previous experience to guide treatment discussions with their physicians

Participants with an infrequent history of uUTI were often satisfied with the treatment discussions with their physicians, attaching little to no emotion to the conversation or prescription, with one participant reporting that “I just got it and took it as advised” (DE09). However, participants with a more frequent history of uUTIs felt that their previous experiences allowed them to have more involved treatment discussions with their physicians, with prescribing decisions guided by past experience with antibiotics. One participant reported that “I was aware that it may not work due to my past experiences” (US22). Conversely, some participants with frequent uUTI reported that they did not always feel as though they were being listened to regarding previous treatments and a plan in moving forwards to other treatments, with one reporting that they “asked if we could try something different to see if it worked better—as soon as I finish antibiotics it comes back very soon afterwards” (US27).

#### Some participants question the need for diagnostic tests

Most participants reported that they had a dipstick test for confirmation of a uUTI. The majority of participants took a pragmatic approach to the need for testing, with an understanding that the test would determine their suitability for antibiotics. One participant reported that “this is standard practice every time I go to my GP” (DE06). Some participants who took a self-management approach mentioned doing an ‘at-home’ test first to confirm infection and the need for treatment. However, some participants with recurrent uUTI questioned the need for diagnostic tests, when they deemed the diagnosis to be obvious.

#### Participants are frustrated by the burden of time needed for visits to their physicians

In addition to frustration in requiring a positive diagnostic test, some participants reported frustration at the burden of time needed for visits to their PCP. Participants with recurrent uUTI felt they knew when they had another uUTI, and therefore felt they should be prescribed treatment without the need for a visit to their physician: “It is frustrating that you cannot call the doctor and get the prescription based on your reporting of symptoms and knowledge that you have a uUTI” (US36).

### Treatment failure causes frustration and anger

#### Trying to understand treatment failure

Among participants who experienced treatment failure, most reported going back to the same physician or clinic as they did for initial treatment. In explaining treatment failure, some physicians provided a basic explanation of bacterial resistance. Participants were generally relieved to have an explanation for treatment failure and motivated to receive a second antibiotic to resolve symptoms. One participant said, “Bacteria are too stubborn. I didn’t really ask for much detail, I just took [the antibiotic] because I wanted the UTI to go away as quickly as possible” (DE01). When the physician offered no explanation for the treatment failure, participants sometimes felt responsible and blamed themselves. In the words of one woman, “I was asking myself whether I did something wrong: sitting on a cold surface, not drinking enough…” (DE03).

#### Emotional reactions to treatment failure

Participants who experienced treatment failure described many feelings, including helplessness that the antibiotic was not working, frustration at “going round in circles,” disappointment that treatment did not work, irritability because nothing seemed to be working, anger at having to go to the doctor again (which was seen as inconvenient), exhaustion with continuing symptoms, and worry (“Is something underlying?” “Is this my fault?”). One participant said, “You’re irritable. You don’t want to be bothered. You’re taking pills and nothing seems to be working. And you’re not sleeping … Sometimes I just feel like I’m going around, and around and around and around” (US22). When offered a second antibiotic, participants described feelings of worry and concern about whether the new treatment would work, as well as frustration and annoyance at having to take another round of antibiotics. A participant said, “I cried in the practice, because I was frustrated and in despair” (DE02).

#### Similarity of feelings about recurrence and treatment failure

There was substantial overlap in the emotional experience of uUTI recurrence and treatment failure of an acute episode. With both recurrence and treatment failure, participants experienced the same feelings of continual suffering, as well as the same experience of having to continually revisit the doctor. Participants described feelings of frustration at suffering from yet another uUTI impacting their life and routine; helplessness because of a “hamster wheel” effect of continually suffering and visiting the doctor; dread and anxiety because of the expectation of another uUTI, as well as anticipation of the negative symptoms to come; and worry that there was an underlying issue causing treatment failure or recurrence (Fig. 1).

### Prospect of recurrent uUTIs provokes dread and helplessness

#### Fear of the next uUTI

After resolution of a uUTI episode, participants continued to experience residual feelings.

Anticipation of the next uUTI and the pain and disruption it would bring caused dread and a feeling of helplessness in participants with frequent uUTI. Indeed, one woman described this feeling as, “Here we go again” (US42). Participants who suffered very frequent uUTIs expressed helplessness that the problem kept returning and anxiety at the prospect of another infection being just around the corner. Participants became anxious about suspected triggers of uUTIs, such as a new sexual partner, restarting birth control pills, giving birth, stressful life experiences, and even cool weather. Some participants described feeling helpless that they are in a permanent cycle, waiting for the next uUTI. A participant said, “I felt trapped. Like I couldn’t break free or away from it. I was trapped in a cycle of having these UTIs back to back and I couldn’t seem to break free” (US32).

#### Loss of trust in physicians

Participants who experienced recurrent uUTIs sometimes raised concerns with the physician, and they expressed a lack of trust in their HCP if requests were not responded to. Women experienced anxiety and anger that treatment was not working or that further problems were not investigated. One participant said, “I asked if we could try something different to see if it would work better. In the past, as soon as I finished the antibiotics, the problem came back very soon afterwards” (US27). Some participants described not feeling they were “heard” regarding previous treatments and their desires for the management plan moving forward, which led to frustration with and distrust in their HCP. Belief in the physician was questioned, and some participants considered changing PCPs or visiting a specialist. One woman said, “I do not have high expectations from medical professionals—doctors have missed things before” (US19).

#### For some, new feelings of resourcefulness

In contrast, a few participants described positive developments after having experienced recurrent uUTIs. Some received specialist referrals after repeated treatment failure or recurrence, and these participants expressed relief at undergoing further investigations. A few participants suffering from recurrent uUTIs mentioned feelings of positivity when they found ways of keeping the problem controlled. A participant said, “I did recover faster because I was drinking more water, so now I feel like I’ve got a little more empowerment every day to prevent them” (US46).

## Discussion

In this qualitative, interview-based study of participants who experienced uUTIs (including recurrent uUTIs) in the past year, women described a striking range of emotions related to the burden of symptoms and to their experiences of recurrent infection and apparent treatment failure. Women described frustration as symptoms interfered with typical life activities and relationships, and they felt hopeless and fearful in the context of recurring infections.

Our findings have some correlation with previous research on the experiences of participants with uUTIs. Similar to the qualitative interview study by Eriksson et al. [13] of Swedish women with recurrent uUTIs, women in this study described a range of effects of uUTIs on their daily lives, relationships, and sleep, along with negative emotional reactions to these experiences. Our study also uncovered an additional realm of emotions involving helplessness and dread in the context of treatment failures and recurring infections, and a lack of understanding in the need for diagnostic tests and frustration in the burden of time needed for visits to physicians. Like the work of Leydon et al. [22], this analysis found that some women blamed themselves for treatment failure and recurrent uUTIs. There is a crucial need for accurate information to be given to participants, explaining that the issue of antibiotic resistance lies with the infecting organism and not the patient.

The finding that the financial impact of uUTIs is often greater for US than German participants is not surprising. In the US, many patients are uninsured or have high-deductible insurance plans and limited coverage for prescription drugs, so their out-of-pocket expenses for medical visits, urine testing, and antibiotics can be substantial. Additionally, many US workers do not get paid time off for medical appointments and sick leave. In Germany, patients pay only a small copayment for antibiotics, although they do pay out-of-pocket for phytopharmaceuticals and over-the-counter pain relievers. The impact of missed work on earnings is low in Germany because sick leave is routinely paid by employers.

Patient education strategies should highlight the need to help patients understand why an office visit and urine testing may be needed, especially for recurrent uUTIs. Patients want greater reassurance around their antibiotic resistance concerns, and they want to be better informed about the possible side effects of antibiotic treatment. Previous research has demonstrated that physicians and patients often have different perceptions of uUTI symptom severity and that improved communication between physicians and patients can improve health outcomes [23]. Shared decision-making interventions and efforts to provide information to patients via printed “after visit summaries” or other patient-doctor communication tools (leaflets, videos, smartphone apps, etc.) can help maximize patient empowerment and satisfaction with care [24]. Over the course of the interviews, participants offered the following suggestions to improve patients’ experience of uUTI. We include our participants’ comments in Table 3, along with our own thoughts.

**Table 3:** Suggestions from participants

Participant comments	Authors’ notes
Participants wanted to improve their knowledge of how to reduce the likelihood of a uUTI, their awareness of symptoms, and their understanding of when to seek medical intervention	We hope all clinicians welcome patients’ desire for better understanding of this common condition, addressed through the patient consultation
Participants wanted more accurate antibiotic prescribing, which they felt would avoid the frustration of losing time during initial unsuccessful treatment. Participants believed better testing would allow physicians to choose an effective antibiotic for initial treatment	Patients with risk factors for a uUTI caused by an antibiotic-resistant organism may require a different empirical approach. Conducting a urine culture and susceptibility test for appropriate patients more likely to have a uUTI caused by an antibiotic-resistant pathogen before empirical treatment will help appropriately guide subsequent treatment. In addition, prior culture results (i.e., speciation, susceptibilities) and local outpatient-specific antibiograms can help guide treatment for UTI [25]
When communicating with their HCP, participants wanted to be better informed about the possible antibiotic side effects and they wanted better understanding of antibiotic resistance as it applies to their own infection	These requests further illustrate the importance of providing patient education at each visit
Participants desired greater efficiency in HCP interactions. In particular, they wanted an easier and more convenient way to obtain a prescription, ideally over the phone if the infection is recurrent or previously treated	In some clinics, resistance to first-line antibiotics is high, so urine culture is needed to prescribe the appropriate antibiotic. Approaches can be tailored to patient circumstances, but our participants’ comments again highlight the need to educate patients about why an office visit and urine testing may be needed

*DE* Germany, *HCP* healthcare professional, *US* United States, *uUTI* uncomplicated urinary tract infection

One limitation of this study is that participants were drawn from a variety of settings in Germany and the US and are not necessarily representative of all women experiencing uUTIs. We cannot exclude a selection bias toward women with a higher symptom load and those who perceive a greater impact of UTI on their quality of life. Another limitation is there is potential for participation bias due to the need to downloading an app, which could exclude participants with older mobile phones or who did not feel technologically capable of completing the exercise. Sociodemographic information about participants (income, education, geographic distribution, urban/rural setting, race, etc.) was not collected, which also makes it difficult to assess whether our findings are transferable to larger populations. Differences between the US and German patient samples may have affected study findings. On the other hand, the relatively large number of participants (for a qualitative interview-based study) and inclusion of participants from both Europe and North America provided a broad perspective on participants’ experiences in these geographic regions. A further strength of this study is that it used participants from both the US and Germany, which allowed for the comparison of experiences of women from two different healthcare systems. There was a high degree of consistency in experiences, with financial impact being the key difference between the participants of the two countries.

## Conclusion

In summary, this analysis points to measures clinicians can take when interacting with participants with uUTIs. Considering patients’ reports of isolation and embarrassment, clinicians can help patients with uUTIs understand how common the condition is. Patient education should focus on explaining key aspects of the pathophysiology of uUTIs; that treatment failure lies with the infecting organism, not the patient; that recurrent uUTIs can happen even without underlying medical or anatomic conditions; and that urine testing is necessary in certain situations.

## Supplementary Information

**Additional file 1**. Text of structured interview, including instructions to the moderator conducting interview.

## Data Availability

The datasets generated during and/or analyzed during the current study are not publicly available due to patient confidentiality. Anonymized datasets used and/or analyzed during the current study are available from the corresponding author on reasonable request.

## Abbreviations

AKEK: Association of Medical Ethics Committees
DE: Germany
EphMRA: European Pharmaceutical Market Research Association
HCP: Healthcare provider
IRB: Institutional Review Board
US: United States
UTI: Urinary Tract Infection
uUTI: Uncomplicated Urinary Tract Infection
